# Biomechanical effects of rocker shoes on plantar aponeurosis strain in patients with plantar fasciitis and healthy controls

**DOI:** 10.1371/journal.pone.0222388

**Published:** 2019-10-10

**Authors:** Christian Greve, Dorianne Schuitema, Bert Otten, Laurens van Kouwenhove, Erik Verhaar, Klaas Postema, Rienk Dekker, Juha M. Hijmans

**Affiliations:** 1 University of Groningen, University Medical Center Groningen, Department of Rehabilitation Medicine, Groningen, The Netherlands; 2 University of Groningen, University Medical Center Groningen, Center for Human Movement Science, Groningen, The Netherlands; 3 Radboud University, Donders Institute for Brain, Cognition and Behaviour, Nijmegen, The Netherlands; University of Colorado Boulder, UNITED STATES

## Abstract

Plantar fasciitis is a frequently occurring overuse injury of the foot. Shoes with a stiff rocker profile are a commonly prescribed treatment modality used to alleviate complaints associated with plantar fasciitis. In rocker shoes the apex position was moved proximally as compared to normal shoes, limiting the progression of the ground reaction forces (GRF) and peak plantarflexion moments during gait. A stiff sole minimizes dorsiflexion of the toes. The aim of this study was to investigate whether the biomechanical effects of rocker shoes lead to minimization of plantar aponeurosis (PA) strain during gait in patients with plantar fasciitis and in healthy young adults. 8 patients with plantar fasciitis (1 male, 7 females; mean age 55.0 ± 8.4 years) and 8 healthy young adults (8 females; mean age 24.1 ± 1.6 years) participated in the study. Each participant walked for 1 minute on an instrumented treadmill while wearing consecutively in random order shoes with a normal apex position (61.2 ± 2.8% apex) with flexible insole (FN), normal apex position with stiff insole (SN), proximal apex position (56.1 ± 2.6% apex) with flexible insole (FR) and proximal apex position with stiff insole (SR). Marker position data of the foot and lower leg and GRF were recorded. An OpenSim foot model was used to compute the change in PA length based on changes in foot segment positions during gait. The changes in PA length due to increases in Achilles tendon forces were computed based on previous data of a cadaver study. PA strain computed from both methods was not statistically different between shoe conditions. Peak Achilles tendon force, peak first metatarsophalangeal (MTP1) joint angle and peak plantarflexion moment were significantly lower when walking with the rocker shoe with a proximal apex position and a stiff insole for all subjects (p<.05). Changes in Achilles tendon forces during gait accounted for 65 ± 2% of the total PA strain. Rocker shoes with a stiff insole reduce peak dorsiflexion angles of the toes and plantar flexion moments, but not PA strain because the effects of a proximal apex position and stiff insole do not occur at the same time, but independently affect PA strain at 80–90% and 90–100% of the stance phase. Rocker shoes with an apex position of ~56% are insufficient to significantly reduce peak PA strain values in patients with plantar fasciitis and healthy young adults.

## Introduction

Plantar fasciitis is a frequently occurring overuse injury of the foot. Approximately 10% of the general population experiences complaints associated with plantar fasciitis once in their life [1]. Plantar fasciitis is in the top five most frequently occurring overuse injuries in runners, but it is seen in both athletic and non-athletic populations [2–4].

Patients suffering from plantar fasciitis experience mostly pain along the proximal part of the plantar aponeurosis and around its attachment on the calcaneal tuberosity. Pain aggravates with weight bearing such as standing and walking. Despite a wide range of treatment modalities [5–7] plantar fasciitis often reaches a chronic state impairing community ambulation and participation in work, sport and leisure time activities [8,9]. Overuse through high mechanical stress and repetitive micro traumata play an important role in the development and persistence of plantar fasciitis [10].

The plantar aponeurosis (PA) or planta fascia is a strong fibrous band which originates at the calcaneus and extends distally as five separate bundles to the phalanges [11]. At the calcaneus, the PA is directly connected to the paratenon of the Achilles tendon through the periosteum of the heel [12]. During gait, PA length increases when the heel is off the ground through the posterior movement of the calcaneus [13] and from mid stance to heel-off through the pulling force of the Achilles tendon [11,14]. At the end of the stance phase, increases in dorsiflexion of the metatarsophalangeal (MTP) joints further increase PA length [13–17]. Changes in PA length during gait (PA strain) increase mechanical load of the PA and in case of pathology can lead to pain and discomfort [18,19].

A frequently used treatment modality to lower PA loading during gait is a stiff rocker shoe with a proximal apex position. A proximal apex position and stiff insole limits the progression of the center of pressure distally to the front of the foot during gait. A more proximal point of application of the ground reaction forces requires lower peak plantarflexion moments and therefore Achilles tendon forces during push-off [20]. In addition, minimizing bending of the shoe with a stiff insole prevents excessive dorsiflexion of the toes during pre-swing [17]. However, until now it remains to be established whether these biomechanical effects associated with a proximal apex position and stiff insole lead to smaller PA strain and hence mechanical load during gait in patients and healthy controls [21].

The aim of the current study was to investigate the effect of a more proximal apex position and a stiff insole on PA strain during gait in patients with plantar fasciitis and healthy young adults. To the best of our knowledge, this will be the first time addressing the effect of increases in Achilles tendon forces separate from the effect of MTP dorsiflexion angles on total PA strain during gait using data from a cadaver study in combination with a musculoskeletal model [13]. The total PA strain was computed as the sum of the change in PA length due to changes in MTP dorsiflexion angles and the change in PA length due to Achilles tendon forces. Finally, orthotic treatment recommendations are provided based on the acquired results. We hypothesized, based on previous studies, that the mechanical load of the PA is lowest in patients with plantar fasciitis and healthy young adults when walking with a more proximal apex position and a stiff insole [13,14,17,20,22]. Because complaints associated with plantar fasciitis aggravate with weight bearing such as standing and walking [23], patients with plantar fasciitis might adapt the way they walk and use smaller steps as compared to healthy young adults. These adaptations in spatio-temporal parameters might affect the theoretical effectiveness of rocker shoes on reducing plantar fascia strain during gait. To account for this possible interaction between patient group and effectiveness of the shoe adaptation we used healthy young adults as a control group.

## Methods

### Participants

Healthy young adults were included for participation if they were older than 18 years and free of any self-reported musculoskeletal disorder negatively affecting gait. Patients eligible for inclusion also had to be at least 18 years old and free of any self-reported musculoskeletal disorder, but were also diagnosed with plantar fasciitis by a rehabilitation physician. The study was conducted according to the principles expressed in the declaration of Helsinki [24]. All participants provided written informed consent prior to the experiment. This study was approved by the medical ethical review board of the University Medical Center Groningen, The Netherlands (METc 2016.087).

### Experimental set-up

**Fig 1:**
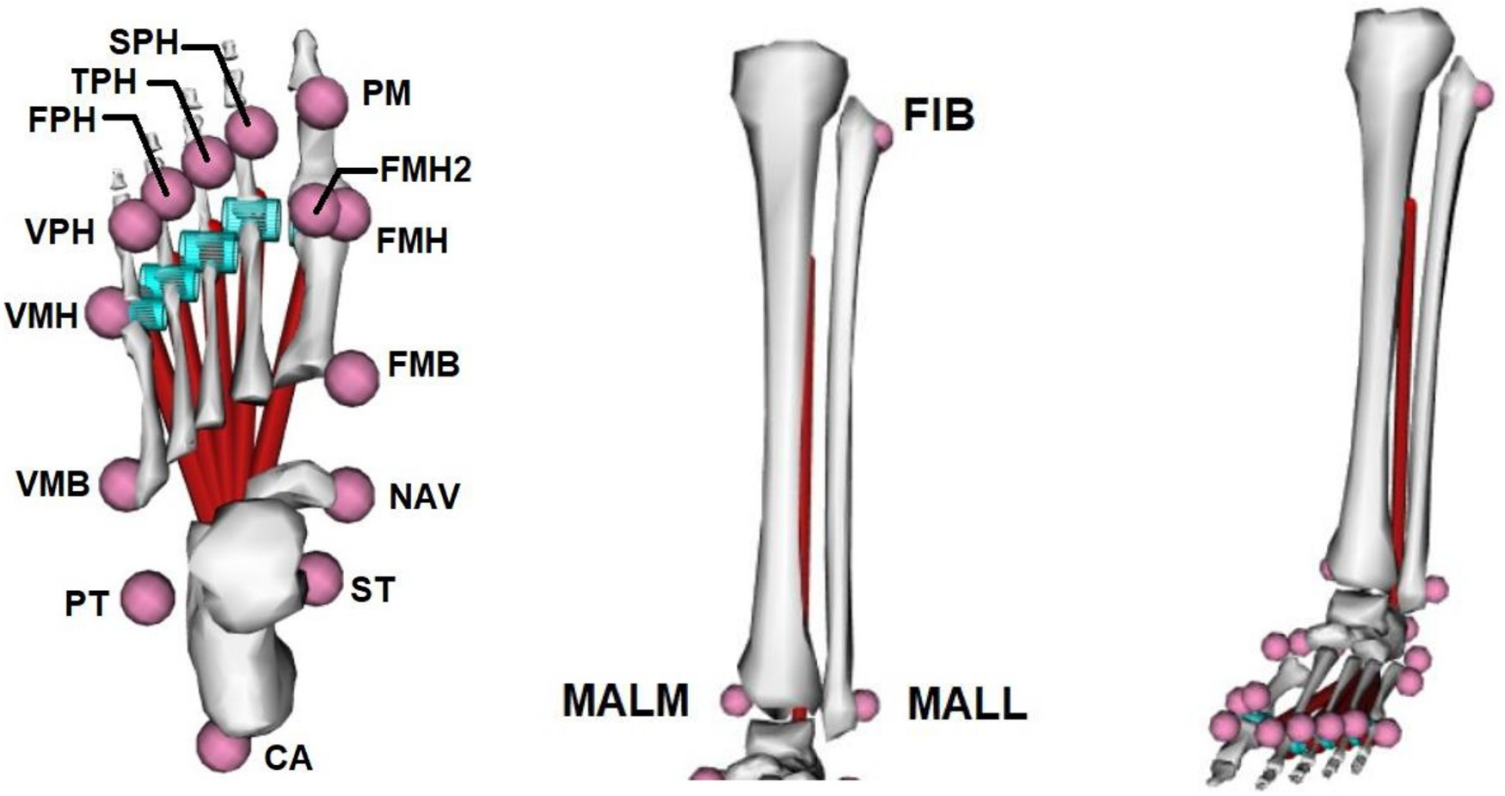
Foot model and marker positions. CA = calcaneus, ST = sustentaculum tali, PT = peorenal tubercle, NAV = navicularis, FMH, FMH2 and VMH = first and fifth metatarsal head, FMB and VMB = first and fifth metatarsal base, PM = head of the proximal phalanx of the 1st toe, SPH = head of the proximal phalanx of the 2nd toe, TPH = head of the proximal phalanx of the 3rd toe, FPH = head of the proximal phalanx of the 4th toe, VPH = head of the proximal phalanx of the 5th toe, MALM = medial malleolus, MALL = lateral malleolus, FIB = fibula.

The study was conducted at the Gait Real-time Analysis Interactive Lab (GRAIL) (Motekforce Link B.V., Amsterdam, The Netherlands) of the Department of Rehabilitation Medicine of the University Medical Center Groningen, The Netherlands. The GRAIL consists of a dual-belt treadmill with a force plate under each belt, a 180 degrees projection screen and 10 motion capture camera’s (Vicon Bonita 10) controlled by Vicon Nexus 2.6 software (Oxford Metrics, Oxford, United Kingdom). D-flow 3.28.0 Treadmill Module (Motekforce Link B.V., Amsterdam, The Netherlands) was used to control treadmill speed and the screen projection. All participants walked in the same virtual reality environment at their self-selected walking speed. Optical flow was synchronised to treadmill speed. Position data of 17 retroreflective markers (10 mm diameter) placed at the right or left foot and tibia was recorded at 100 Hz. The dominant leg (self-reported) was measured. The markers were placed on the calcaneal tubercle, sustentaculim tali, peroneal tubercle, navicularis, first and fifth metatarsal head and base, medial and lateral malleolus, fibula and on the (proximal) interphalangeal joint of each toe (adapted from Caravaggi et al. [25]) (Fig 1). To prevent marker occlusion and improve visibility, the markers on the calcaneus and the first and fifth metatarsal base (PT, ST, FMB, VMB) were placed on a 2 cm rod. GRF data were recorded at 1000 Hz and synchronised with motion capture data. Force plates baselines were reinitialized prior to each trial.

### Experimental procedure

Before the start of the experiment, participants walked in their own shoes for 6 minutes on the treadmill to get acquainted to the treadmill and to determine the comfortable walking speed [26]. During those 6 minutes, participants were regularly asked to indicate whether the actual speed was their comfortable speed or whether they would like to walk slower or faster. The comfortable walking speed was used for all experimental conditions.

After marker placement a static reference measurement was conducted with the foot in normal unloaded position. During the static measurement participants stood on the contralateral leg and placed their foot on a paperboard box to facilitate unloading. The angle between tibia and foot in the sagittal plane was approximately 90 degrees. The examiner checked whether the foot was unloaded through visual inspection of the real-time projection of the magnitude of the vertical GRF. If load on the foot exceeded approximately 10–15% of the body weight participants were instructed to relieve the load on the foot. The static measurement was repeated for each shoe condition and was used to determine the resting length of the PA. During the experimental condition subjects walked at their comfortable speed for 1 minute in each condition. Participants were instructed to keep each foot on a separate belt and walk like they normally would without using the handrails.

### Experimental conditions

**Fig 2:**
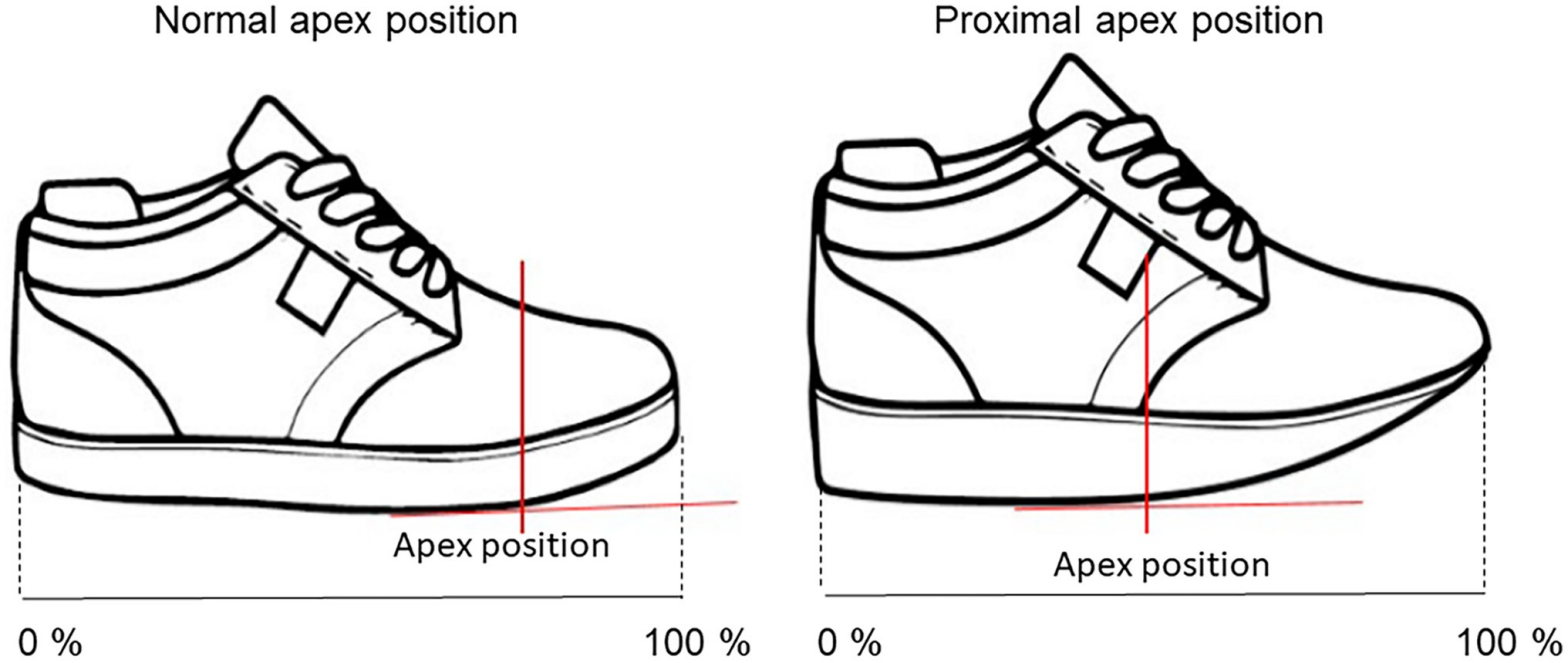
Illustration of a shoe with a normal and proximal apex position. Total shoe length is indicated as percentage. The proximal apex position is at ~50% of total shoe length.

**Fig 3:**
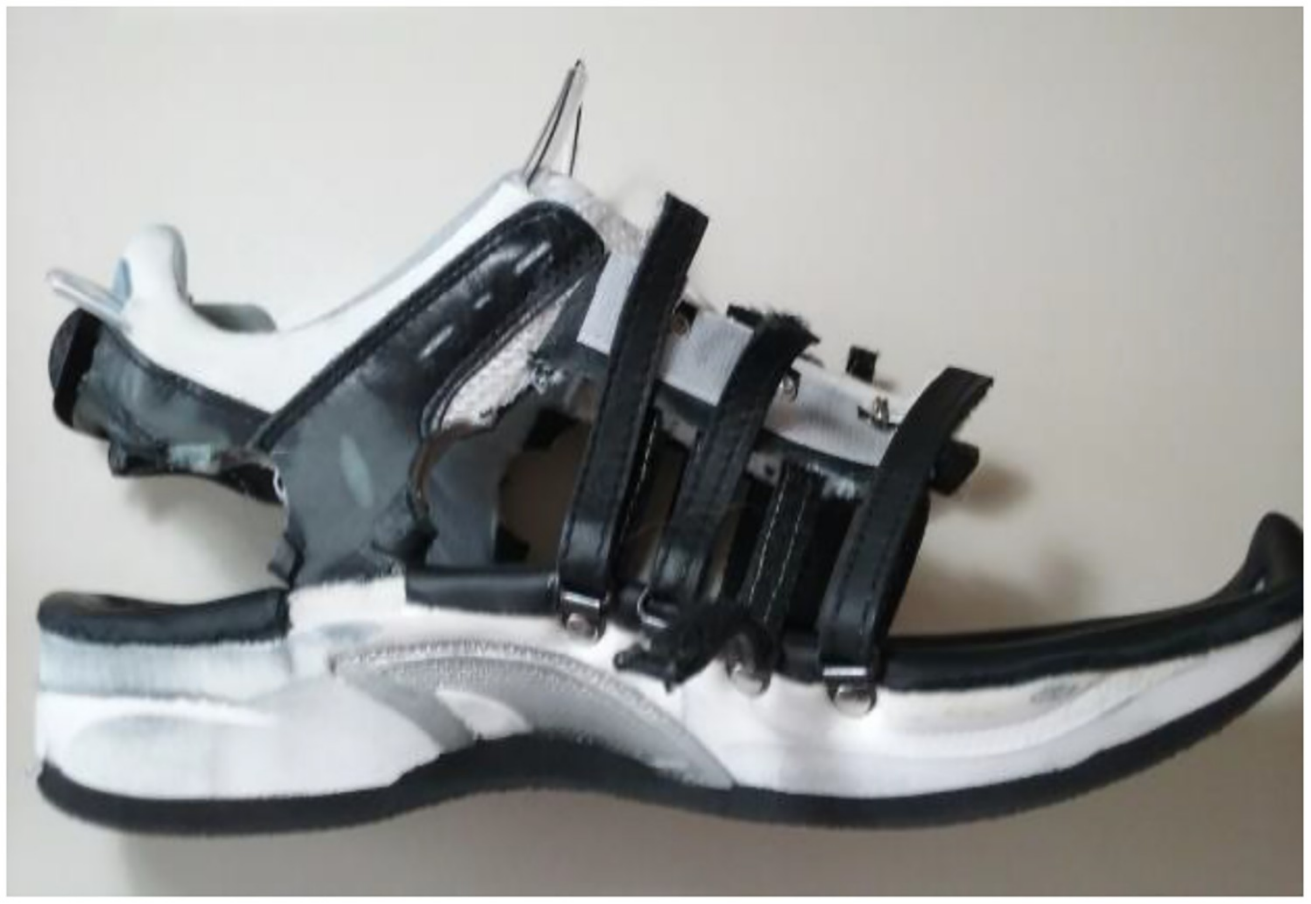
Experimental shoe.

To test the hypotheses, participants walked with four different shoe types: 1) shoe with a flexible insole and normal (61.2 ± 2.8%) apex position (FN), 2) shoe with a flexible insole and proximal (56.1 ± 2.6%) apex position (FR), 3) shoe with a stiff insole and normal apex position (SN) and 4) shoe with a stiff insole and a proximal apex position (SR). The position of the proximal apex was defined based on clinical expert experience. The stiffness of the shoe with a carbon fiber was 102.2 Nm/rad and the stiffness of the shoe without carbon fiber was 18.2 Nm/rad. A detailed explanation of the testing procedure is provided as appendix (S1 Text). After preparation of the different shoes, the apex percentage was calculated as the distance between the heel and the apex position along the midline of the shoe with respect to total shoe length (Fig 2). The stiffness of the insole was manipulated by replacing the flexible insole with a carbon fiber insole. The apex angle was 85% for all shoe conditions. To manipulate the apex position, two different shoe types were used with a proximal and normal apex position. The shoes were adapted to place the markers on the bony landmarks but still provide sufficient support for the foot (Fig 3). The shoe adaptations were made by a certified orthopaedic technician. Testing order of the shoe conditions was randomised via MATLAB’s random number generator (version 2016b, MathWorks, Inc., USA). Participants were blinded to the intervention.

### Data analysis

A custom made foot model (Fig 1) was used in Vicon Nexus 2.6 (Vicon Motion Systems Ltd, Oxford, UK) to record marker positions during the experiments. Custom made MATLAB scripts were used to process the motion capture and force plate data. Force plate data were down sampled to 100 Hz and together with the motion capture data filtered with a 4^th^ order low-pass Butterworth filter with a cut off frequency of 6 Hz. Data analysis focused on the stance phase of the gait cycle. Heel strike and toe off were determined using center of pressure data from both force plates as described by Roerdink et al (2008) [27,28]. The first 6 frames (60 ms) after initial contact were excluded from the analysis due to heel marker occlusion. In addition, to improve data quality, steps where participants walked with two feet on the same belt and steps containing over 10 gaps in the marker position data were removed from the analysis. Remaining gaps were filled with a 1^st^ order polynomial interpolation. Each gait cycle was visually scrutinized on accuracy of event detection before being used for further analysis. Step length was computed as the difference between the anterior-posterior position of the center of pressure between the left and right leg at initial contact.

The length of the PA was computed using a musculoskeletal model in OpenSim (version 3.3) similar to previous studies [25,29,30]. The OpenSim model consisted of 10 rigid bodies: lower leg, talus, calcaneus, navicularis, metatarsals and five phalanges. The tibia and fibula were represented in one segment and were connected to the talus via a one degree of freedom (DOF) ankle joint, allowing plantar and dorsal flexion. The subtalar joint connects the talus to the calcaneus, providing inversion and eversion. All metatarsals were modelled as one segment with 6 DOF to simulate flexibility of the midfoot. Unlike the metatarsals, all phalanges were modelled separately. Each phalange was connected to the metatarsal segment via a one DOF joint, allowing flexion and extension. The PA was simulated as 5 separate bands all having their origin in the calcaneus and extending distal to one of the proximal phalanges. OpenSim wrapping cylinders were used to simulate wrapping of the PA around the metatarsal heads. The soleus muscle was also incorporated in the model to determine the moment arm of the Achilles tendon based on the inverse kinematic analysis output in OpenSim [30]. The soleus had its origin at the lateral side of the tibia, at one fifth of the distance from the proximal (FIB) and distal fibula (MALL) marker and its insertion at the proximodorsal end of the calcaneus. Before performing OpenSim simulations, the model was scaled for each participant and shoe condition individually according to the corresponding static trial. The scaling was visually checked and if needed corrected by the examiner.

Strain of the PA was calculated in three subsequent steps. First, the length of all five PA bundles and the moment arm of the soleus muscle were calculated via the inverse kinematics tool, inverse dynamics tool and analysis tool in OpenSim. Second, the strain in the PA based on kinematic analysis (ε_pos_) was calculated as:

$$\varepsilon_{pos}=\frac{l-l_{rest}}{l_{rest}}$$ (1)

where *l* is the measured length of the PA during gait and *l*_*rest*_ the rest length obtained from static unloaded trials. In the next step the 3D ankle joint moment (*τ*_*ankle*_) was computed as the cross product between the ankle joint moment arm and the magnitude of the ground reaction force vector:

$$\tau_{ankle}=r_{Ankle}\;x\;GRF$$ (2)

with *r*_*Ankle*_ representing the perpendicular distance between the GRF vector and ankle center of rotation (GRF moment arm). The ankle joint center of rotation was approximated as 50% of the distance between the lateral and medial malleolus. The sagittal plane ankle joint moment (plantarflexion moment; *τ*_*anklePf*_) was used for further analyses and to estimate Achilles tendon forces during gait:

$$F_{ach}=\frac{\tau_{anklePf}}{r_{ach}}$$ (3)

where *r*_*ach*_ is the moment arm of the Achilles tendon.

Finally, the effect of Achilles tendon force on PA strain was added by making use of data provided in a previously published cadaver study [31]. Carlson et al (2000) described the relation between changes in Achilles tendon force and PA strain by measuring the change in PA length after increasing Achilles tendon forces in a cadaver foot with the first MTP joint fixed at 0, 15, 30 and 45 degrees dorsal flexion [31]. We approximated the relationship between increases in Achilles tendon forces and PA strain with the data provided in table 1 of Carlson et al (2000) [31]. First, the values in each column of table 1 were subtracted by the corresponding strain values of 0 N Achilles tendon force. Next, the PA strain values of the Achilles tendon forces 100, 200, 300, 400 and 500 were averaged across MTP joint angles. This data was finally used to determine coefficients a and b of a second order polynomial with a least square algorithm:

$$\varepsilon_{ach}=a∙F_{ach}+b∙\sqrt{F_{ach}}$$ (4)

Eq 4 was used to estimate the change in PA strain due to changes in Achilles tendon forces during gait (ε_ach_). *F*_*ach*_ represents the force on the Achilles tendon. Coefficients a and b were determined with the in-build matlab function polyfitzero, yielding a = 1.5062 · 10^−5^ and b = 7.9032 · 10^−4^. The PA strain obtained from the kinematic analysis was added to the PA strain from the change in Achilles tendon force to obtain total PA strain (Ɛ_total_):

$$\varepsilon_{total}=\varepsilon_{pos}+\varepsilon_{ach}$$ (5)

MTP1 contributes relatively more than the other MTP joints to strain on the PA [32,33]. Therefore, in the following analyses we focused on the effects of the different shoe conditions and participant groups on the strain of the first bundle of the PA.

### Statistical analysis

Statistical analysis was performed using SPSS version 25. To determine the effect of a proximal apex position and a stiff insole on peak PA strain, peak Achilles tendon force, peak plantarflexion moment and peak MTP1 angle averaged across gait cycles in patients with plantar fasciitis and healthy young adults repeated measures ANOVA were performed. In addition, a repeated measures ANOVA on MTP dorsiflexion angles at the moment of peak Achilles tendon forces was used to establish whether MTP dorsiflexion angles differed between shoe conditions at the moment of peak Achilles tendon forces. Finally, we statistically analyzed the effect of the different shoe conditions on step length. For the repeated measures analyses, the between subjects factor was group (patients vs control) and the within subjects factor was shoe condition (FN, SN, FR, SR). For the analysis of the effect of Achilles tendon force on PA strain an extra within subjects factor was added (with Achilles tendon force, without Achilles tendon force). Bonferroni corrected tests were used for post-hoc analysis. Differences were considered significant at p<.05. To interpret the significant main effects of the ANOVA’s, the generalized eta-squared (η^2^_G_) for effect size was used [34,35]. The effect sizes were interpreted according to Cohen’s (Cohen 1988) recommendation of .02 for a small effect, .13 for a medium effect and .26 for a large effect [35].

## Results

### Participant characteristics

In total nine patients and ten healthy young adults participated in the study. Due to technical reasons force data were not available for all conditions of three participants. Therefore three participants were excluded from the results. Participant characteristics are given in Table 1. A significant difference was found for age. On average 40 ± 16 gait cycles were used for the statistical analysis in each shoe condition and participant.

**Table 1:** Participant characteristics (mean ± std).

	PF	CG
n	8	8
Age	55.0 ± 8.4*	24.1 ± 1.6*
Gender (M/F)	1/7	0/8
Height (m)	1.73 ± 0.03	1.75 ± 0.02
Weight (kg)	77.4 ± 13.9	68.8 ± 5.9
Measured side (L/R)	4/4	1/7
Walking speed (km/h)	4.1 ± 0.61	4.1 ± 0.53

*p<.05

CG: control group; PF: plantar fasciitis group

**Fig 4:**
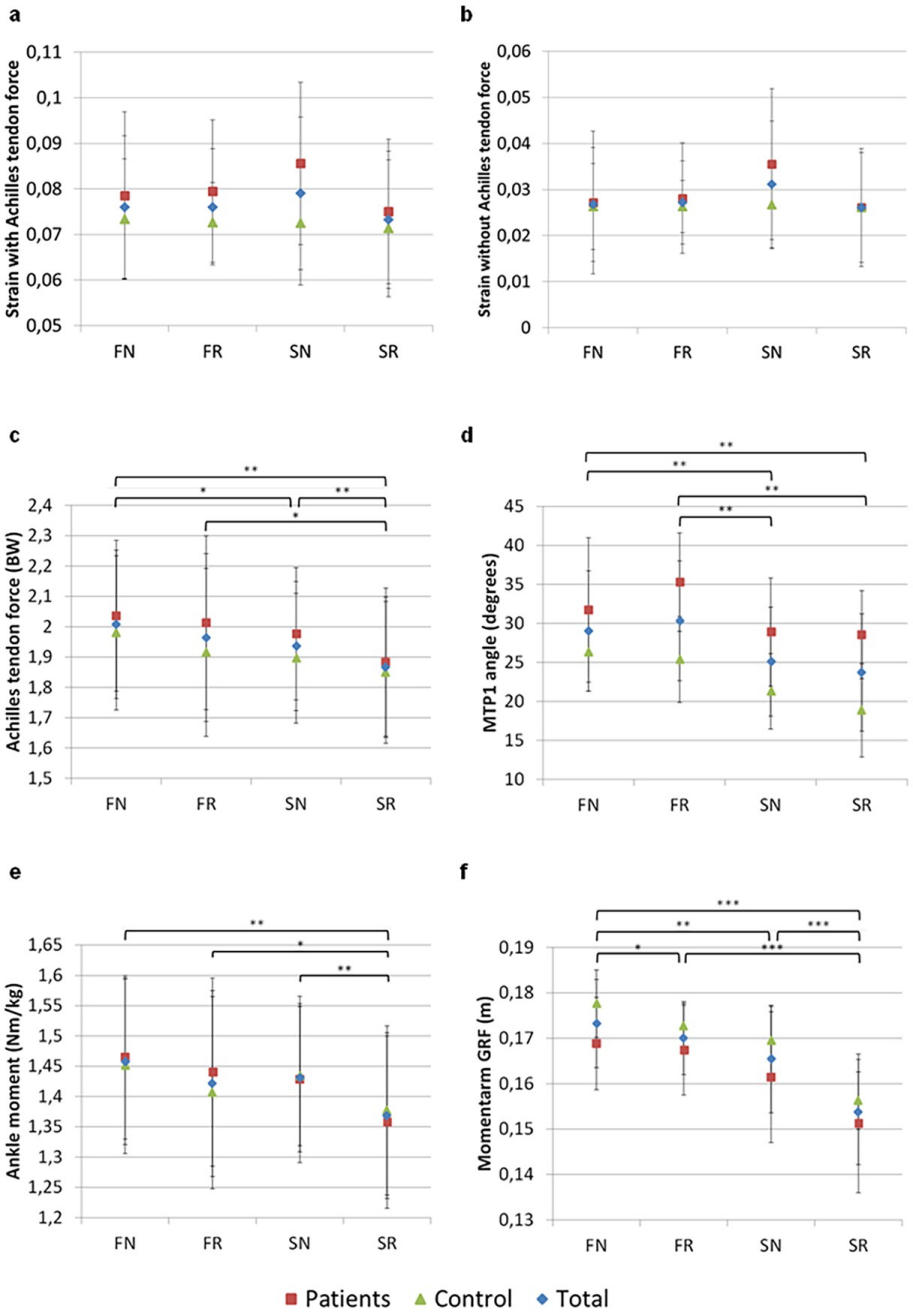
Effects of shoe conditions on biomechanical variables. Average values for PA strain with (a) and without (b) Achilles tendon force, MTP1 angle (c), Achilles tendon force (d) and plantarflexion moment (e) at peak loading and moment arm of GRF at heelrise (f) (*p<.05; **p<.01; ***p<.001).

**Fig 5:**
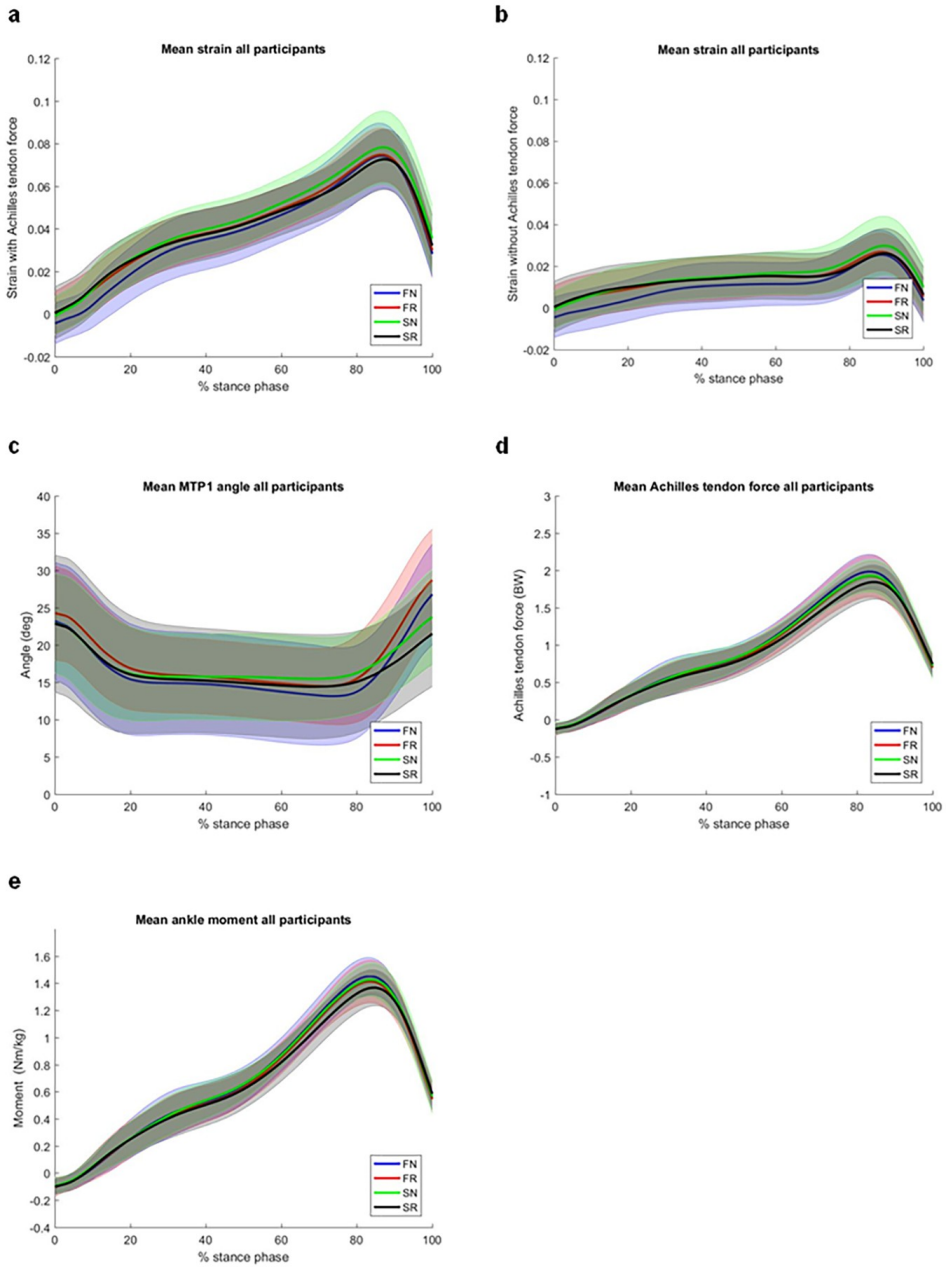
Effects of shoe condition on biomechanical variables during stance phase. Average values for PA strain with (a) and without (b) Achilles tendon force, MTP1 angle (c), plantarflexion moment (d) and Achilles tendon force (e) per condition during stance phase for all participants. Shading indicates between subjects standard deviation.

Peak strain, Achilles tendon force and ankle plantarflexion moments were reported around 80–85% of the stance phase. MTP1 angles peaked at 90–100% of the stance phase. Average peak values of all outcome measures are given in Fig 4 (and S1 Table). Fig 5 shows the temporal profile of the PA strain with and without the effect of Achilles tendon force, MTP1 angle, Achilles tendon force and ankle moment during the stance phase. Note that the first 6 frames after initial contact were removed from the analysis. The temporal profile of our kinematic, kinetic and strain data is comparable to previous studies [17,25,29,36]. The repeated measures ANOVA on step length revealed a significant main effect for shoe condition (p = .045; F_2,27_ = 3.5) showing that both experimental groups walked with slightly smaller steps when walking with a stiff insole and normal apex position (SN = .42 ± .01 meter) as compared to a flexible insole and a proximal apex position (FR = .43 ± .01 meter).

### Effects of rocker shoes on plantar aponeurosis strain

The repeated measures ANOVA on peak PA strain did not reveal a significant main effect for participant group (F(1, 14) = 1.052; p = .322) or shoe condition (F(3, 42) = 2.319; p = .089). Also no interaction was found between participant group and shoe condition (F(3,42) = 1.727; p = .176; Fig 4a).

### Effects of rocker shoes on plantarflexion moment and Achilles tendon force

Both peak Achilles tendon force (F(2.17,30.32) = 11.099; p<.001; η^2^_G_ = .046) and plantar flexion moment (F(2.16, 30.16) = 12.63; p<.001; η^2^_G_ = .058) differed significantly over the shoe conditions. Post-hoc analysis revealed that for both Achilles tendon force and plantar flexion moment SR was significantly lower than FN (ATF: p = .002, PFM: p = .002), FR (ATF: p = .034, PFM: p = .011) and SN (ATF: p = .003, PFM: p = .001). SN was significantly lower than FN for Achilles tendon force (p = .016). No effect was found for participant group (ATF: F(1,14) = 0.313; p = .585, PFM: F(1, 14) = 0.060; p = .810) nor an interaction effect between shoe and participant group (ATF: F(2.17, 30.32) = 0.619; p = .557, PFM: F(2.16, 30.16) = 0.968; p = .397).

### Effects of rocker shoes on MTP1 angle

Patients with plantar fasciitis had larger peak MTP1 dorsiflexion angles than healthy young adults (F(1,14) = 8.375; p = .012; η^2^_G_ = .317). There was no significant interaction effect between participant group and shoe condition (F(3,42) = 1.617; p = .200), but shoe condition did significantly affect peak MTP1 angles (F(3,42) = 14.341; p<.001; η^2^_G_ = .124). Post hoc comparisons revealed that both FN and FR were significantly higher than SN (FN: p = .002, FR: p = .009) and SR (FN: p = .003, FR: p = .001), so in both participant groups MTP1 angle was smaller when wearing stiff insoles instead of flexible ones.

Analysis of the MTP1 dorsiflexion angles at the moment of peak Achilles tendon forces revealed no significant main effect of the shoe conditions or significant interaction effect between shoe conditions and experimental group. In line with the analysis of peak MTP1 dorsiflexion angles, the patient group had significantly larger MTP angles at the moment of peak Achilles tendon forces (p = .034; F_1, 14_ = 5,5; patients = 20.5 ± 1.8, control: 14.4 ± 1.8 degrees).

### Effects of rocker shoes on ground reaction force moment arm

Both participant groups have a comparable moment arm of the GRF at heel off (F(1,14) = 2.188; p = .161). The repeated measures ANOVA revealed a significant main effect for shoe condition on the moment arm of the ground reaction force vector at the ankle joint (F(3,42) = 48.506; p<.001; η^2^_G_ = .349). The moment arm of the GRF was smallest in SR compared to FN (p<.001), FR (p<.001) and SN (p<.001) and largest in FN compared to FR (p = .045), SN (p = .003) and SR (p<.001). There was no significant interaction effect between participant group and shoe condition (F(3,42) = 0.608; p = .613).

### Effect of Achilles tendon force on plantar aponeurosis strain

Changes in Achilles tendon forces during gait accounted for 65 ± 2% of the total PA strain in patients with plantar fasciitis and healthy young adults. The effect of Achilles tendon force was larger in FN (p = .006) and FR (p = .028) compared to SR and FN compared to SN (p = .024), reflected by larger differences between strain calculated with and without the effect of Achilles tendon force.

## Discussion

This study aimed to establish whether a more proximal apex position and a stiff insole are effective in decreasing PA strain during gait in patients with plantar fasciitis. We hypothesized that a more proximal apex position combined with a stiff insole would minimize PA strain through significant reductions in Achilles tendon forces and MTP dorsiflexion angles during gait. Despite a significant reduction in peak Achilles tendon forces and MTP dorsiflexion angles through a proximal apex position and stiff insole, PA strain did not significantly differ between shoe conditions. The effect of shoe adaptations on PA strain in patients with plantar fasciitis were similar as compared to healthy young adults.

Peak Achilles tendon forces occur during push-off at about 80–90% of the stance phase. Because ~65% of total change in PA length is attributed to changes in Achilles tendon forces, peak PA strain values also occur at 80–90% of stance. Hence, minimizing peak Achilles tendon forces through a proximal apex position and stiff insole might minimize peak PA strain values during gait (Figs 4 and 5). However, our statistical analysis revealed that this effect of the rocker shoe on peak PA strain was not significant despite significant reductions in peak Achilles tendon forces. We propose two main reasons for this seemingly paradoxical finding. First, the significant effect of the rocker shoe on Achilles tendon forces was small (η^2^_G_ = .046). Possibly larger reductions in Achilles tendon forces are required to allow the detection of statistically significant reductions in peak PA strain during gait. Second, at the moment of peak PA strain, MTP1 dorsiflexion angles were similar between shoe conditions. Hence, at the moment of peak PA strain (80–90%) only the effect of lower Achilles tendon forces on PA strain (~65% of total PA strain) was considered for our statistical analysis. Similarly, peak MTP1 angles occurred at 90–100% of the stance phase when Achilles tendon forces are low leading to only relatively small reductions in peak PA strain when walking with a stiff insole. Hence, the effects of a proximal apex position and stiff insole on peak PA strain do not occur at the same time, but independently affect PA strain at 80–90% and 90–100% of the stance phase. We argue that rocker shoes with an apex position of ~56% are insufficient to significantly reduce peak PA strain values in patients with plantar fasciitis and healthy young adults during gait.

Somewhat unexpected, patients with plantar fasciitis had significantly larger peak MTP1 angles than healthy young adults independent of shoe condition. Dorsiflexion of the MTP joint during the propulsive phase of gait winds the PA around the head of the metatarsals increasing PA strain. It is therefore possible that the excessive bending of the MTP joint reported in our patient group reflects a risk factor for plantar fasciitis.

### The role of Achilles tendon forces on plantar aponeurosis strain

To our knowledge this is the first study comparing the effect of both Achilles tendon forces and MTP joint dorsiflexion on PA strain during gait. Increases in Achilles tendon force accounted for 65 ± 2% of total PA change. Considering this large contribution of Achilles tendon force on PA strain, one goal of prevention and treatment approaches should always include minimization of peak Achilles tendon forces during gait or sport activities. The rocker shoes used for the current study were made by a certified orthopaedic technician and the position of the proximal apex was defined based on clinical expert experience. Considering that a proximal apex position of 56.1 ± 2.6% did not lead to a significant reduction in PA strain, more aggressive rocker profiles (<56%) should be considered in clinical practice and especially in severe cases of plantar fasciitis. However, it is important to note that lowering the internal plantarflexion moment during gait with a more proximal apex position might lead to higher joint loads at the knee and hip, affect spatio-temporal parameters such as gait speed and step length and the smaller support surface might impair balance [21,37–40]. In addition to orthotic treatment, patients with plantar fasciitis might benefit from gait training aiming to reduce peak Achilles tendon forces during push-off. Especially after recovery from plantar fasciitis, gait training protocols might be considered to prevent reoccurrence of the injury.

## Limitations

In each trial, two different pairs of shoes (rocker and normal) were used, available in three different shoe sizes. The shoes were adapted to allow marker placement. Since many markers had to be placed on the forefoot, that region was least covered by the shoe and therefore toes could move freely. Usually rocker shoes are closed shoes allowing less toe movement. MTP angles are main contributors to total PA strain [31,33], so unnecessary toe movements might have caused larger PA strain due to an increase in MTP angles at initial contact and pre-swing as compared to normal shoes.

The stiffening procedure of the sole of the test shoes deviates from conventional rocker shoes. Conventional rocker shoes usually have a fully stiffened sole, but both test shoes had a soft/flexible sole with a stiff insole that was not fixed to the shoe. Multiple studies were conducted with comparable shoe set-ups [17,41], but it cannot be ruled out that despite the stiff insole, the soft sole is compressed and so the apex might have been shifted distally with weight bearing.

The participants in our study were mainly females. However, since our analysis exclusively used biomechanical measures and we compared the effect of different shoe conditions within subjects we do not expect that gender inequality might have affected our results.

The PA is anatomically connected to the paratenon of the Achilles tendon through the periosteum of the heel [12]. Further evidence for this direct anatomical link stems from previous studies in cadaver feet and living young adults [36,42]. These studies showed that, when the foot is fully on the ground and loaded with body weight, foot segment positions are almost constant [42] but PA strain gradually increases with increases in Achilles tendon forces until push-off [36]. We computed therefore total PA strain as the sum of kinematic changes (MTP dorsiflexion angles and calcaneus movement) and increases in Achilles tendon forces. The effect of Achilles tendon forces on PA strain was modeled based on data from a cadaver study [13]. However, in this study the effect of increases in Achilles tendon forces on PA strain was measured while the calcaneus could move freely [13]. Hence, the data used by Carlson et al (2000) reflects the relationship between PA elongation and increase in Achilles tendon force due to changes in the position of the calcaneus and the direct link of the Achilles tendon with the PA via the periosteum of the heel [12,31]. In our analysis we accounted for the effect of changes in calcaneus position on PA strain in the kinematic analysis. Therefore, adding the effect of increases in Achilles tendon force on PA elongation (Eq 5) based on the data from Carlson et al (2000) might have overestimated total PA strain values once the calcaneus is lifted off the ground at heel-off limiting comparisons of our data to other studies.

Next, the connection between the PA and the Achilles tendon might weaken with advancing age possibly affecting the biomechanical relationship between Achilles tendon forces and PA strain [43]. The analyses performed here are based on data from cadaver studies in young adults. Even though we did not find any relevant and statistically significant differences between the older patients and healthy young subjects, the possibility exists that the model assumptions apply fully to the patient group. Future studies should aim to establish how potential changes in the connection between the Achilles tendon and the PA affect their biomechanical relationship during gait.

Finally, we used a generic foot model scaled to each participants anthropometrics based on marker position data. Individual variations in the diameter of the metatarsal heads, PA origin and insertion were therefore not entirely taken into account and might have affect the absolute values of PA strain. Therefore comparability between our and previous studies with respect to absolute PA strain values might be limited. More individualized musculoskeletal models using magnetic resonance imaging or ultrasound measurements should be considered for future studies to improve model accuracy and comparability between studies. Especially in analysis focusing on between group differences including patients with plantar fasciitis, subject specific anatomy should be taken into account. In addition, incorporating a more realistic connection between the PA and Achilles tendon via the periosteum of the heel bone in musculoskeletal models might improve accuracy of model output during dynamic analyses such as human gait.

## Conclusion

A proximal apex position of 56.1 ± 2.6% in combination with a stiff insole minimizes peak Achilles tendon forces and MTP dorsiflexion angles but not PA strain. Changes in Achilles tendon forces seem to be the main contributor on peak PA strain during gait. Because the rocker shoe modifies two independent kinetic (Achilles tendon force) and kinematic (MTP joint angle) variables at different moments of the stance phase, peak PA strain might remain largely unaffected by rocker shoe adaptations. Treatment and prevention regimes for plantar fasciitis should always include approaches which minimize Achilles tendon forces during gait. In severe cases of plantar fasciitis a proximal apex position of 56.1 ± 2.6% seems insufficient, as no change in PA strain was observed as compared to a normal apex position (61.2 ± 2.8%). Future studies should aim to establish whether more proximal apex positions (< 56%) can lead to significant reductions in PA strain during gait. Furthermore, future studies should consider excessive MTP joint dorsiflexion during gait as potential risk factor for plantar fasciitis.

## Supporting information

S1 Table Average values for PA strain with and without Achilles tendon force, MTP1 angle, Achilles tendon force and plantarflexion moment at peak loading (mean ± std). (DOCX)

S1 Text Stiffness measurements of stiff and normal shoes. (DOCX)
