# Supplementary material for: Biomechanical effects of rocker shoes on plantar aponeurosis strain in patients with plantar fasciitis and healthy controls
Source: PLoS One. 2019 Oct 10;14(10):e0222388. doi: 10.1371/journal.pone.0222388 (PMC6786540; doi:10.1371/journal.pone.0222388)
Supplement: S1 Table — (DOCX) [file pone.0222388.s001.docx]

| Variable | Group | FN | | | | FR | | | SN | | | | SR | | | |  |
| --- | --- | --- | --- | --- | --- | --- | --- | --- | --- | --- | --- | --- | --- | --- | --- | --- | --- |
| N | PF | 8 | | | | 8 | | | 8 | | | | 8 | | | |  |
|  | CG | 8 | | | | 8 | | | 8 | | | | 8 | | | |  |
|  | T | 16 | | | | 16 | | | 16 | | | | 16 | | | |  |
| Strain | PF | .0786 | ± | .0183 | .0795 | | ± | .0156 | | .0856 | ± | .0178 | | .0751 | ± | .0158 | |
|  | CG | .0734 | ± | .0132 | .0726 | | ± | .0088 | | .0725 | ± | .0136 | | .0714 | ± | .0150 | |
|  | T | .0760 | ± | .0157 | .0761 | | ± | .0127 | | .0791 | ± | .0167 | | .0732 | ± | .0150 | |
| Strain – ATF | PF | .0272 | ± | .0155 | .0281 | | ± | .0119 | | .0355 | ± | .0164 | | .0261 | ± | .0128 | |
|  | CG | .0263 | ± | .0093 | .0263 | | ± | .0056 | | .0267 | ± | .0095 | | .0261 | ± | .0119 | |
|  | T | .0268 | ± | .0124 | .0272 | | ± | .0091 | | .0311 | ± | .0137 | | .0261 | ± | .0119 | |
| MTP1 angle (°)^a^ | PF | 31.73 | ± | 9.27 | 35.30 | | ± | 6.32 | | 28.90 | ± | 6.94 | | 28.56 | ± | 5.64 | |
|  | CG | 26.32 | ± | 5.00 | 25.37 | | ± | 5.52 | | 21.30 | ± | 4.84 | | 18.86 | ± | 5.99 | |
|  | T | 29.03 | ± | 7.72 | 30.33 | | ± | 7.69 | | 25.10 | ± | 6.99 | | 23.71 | ± | 7.53 | |
| ATF (BW) | PF | 2.04 | ± | 0.25 | 2.01 | | ± | 0.29 | | 1.98 | ± | 0.22 | | 1.88 | ± | 0.24 | |
|  | CG | 1.98 | ± | 0.25 | 1.91 | | ± | 0.28 | | 1.90 | ± | 0.21 | | 1.85 | ± | 0.23 | |
|  | T | 2.01 | ± | 0.24 | 1.96 | | ± | 0.28 | | 1.94 | ± | 0.21 | | 1.87 | ± | 0.23 | |
| PFM (Nm/kg) | PF | 1.46 | ± | 0.14 | 1.44 | | ± | 0.16 | | 1.43 | ± | 0.14 | | 1.36 | ± | 0.14 | |
|  | CG | 1.48 | ± | 0.14 | 1.44 | | ± | 0.14 | | 1.43 | ± | 0.11 | | 1.40 | ± | 0.13 | |
|  | T | 1.47 | ± | 0.14 | 1.44 | | ± | 0.144 | | 1.43 | ± | 0.12 | | 1.38 | ± | 0.13 | |
| MA GRF (m) | PF | .169 | ± | .010 | .167 | | ± | .010 | | .161 | ± | .014 | | .151 | ± | .015 | |
|  | CG | .178 | ± | .007 | .173 | | ± | .005 | | .169 | ± | .008 | | .156 | ± | .006 | |
|  | T | .174 | ± | .010 | .170 | | ± | .008 | | .165 | ± | .012 | | .154 | ± | .012 | |

ATF: Achilles tendon force (% Bodyweight); CG: control group; MA: momentarm GRF during heel rise; PF: plantar fasciitis group; PFM: plantarflexion moment; Strain - ATF: strain calculated without effect of Achilles tendon force; T: total ; ^a^Positive angle indicates dorsal flexion
