# Supplementary material for: Biomechanical effects of rocker shoes on plantar aponeurosis strain in patients with plantar fasciitis and healthy controls
Source: PLoS One. 2019 Oct 10;14(10):e0222388. doi: 10.1371/journal.pone.0222388 (PMC6786540; doi:10.1371/journal.pone.0222388)
Supplement: S1 Text — (DOCX) [file pone.0222388.s002.docx]

**Method**

To establish the stiffness of the carbon fibre insole and the normal shoes used during the experiment, we used a three-point bending set-up based on previous research ([1–3]). The set-up had a distance between the supporting points (L) of 80 mm (Figure 1) and a stamp connected to a tensile testing machine (DYNA-MESS Prüfsysteme; Stolberg, Germany). The displacement velocity of the stamp was maximal 8.33 mm/s over a distance of 19 mm. This corresponds with a MTP joint angle of approximately 0.87 radians (50 degrees) which is sufficient as the maximal MTP joint angle during gait.


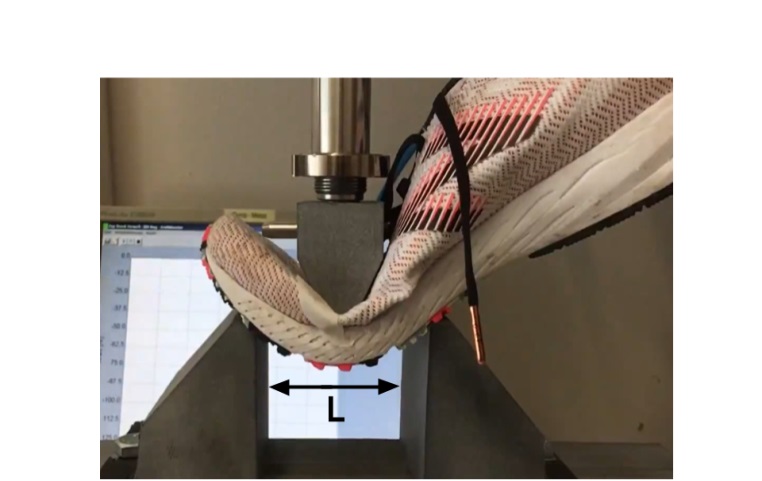


Figure 1. Three-point bending set-up with a 80 mm distance (L) between the supporting points and the stamp placed at 70% of the shoe length (measured from the heel).

Prior to each measurement, a preload is applied to the shoe. This preload assures contact of the stamp with the insole without bending the insole prior to the start of the experiment (figure 2).

**
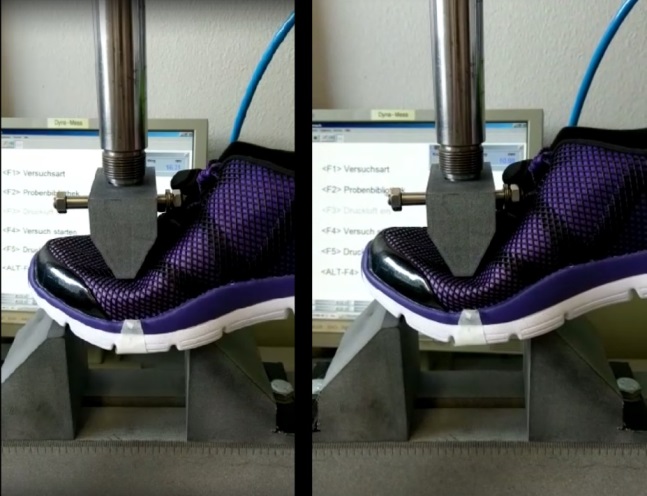
**

Figure 2. Left: no preloading of the shoe. Right: preloading of the shoe. Note that in the right image the shoe sole is not bended.

The flex fulcrum (the position where the stamp should bend the shoe) was placed at 70% of total shoe length. This corresponds with the MTP joint location and is in accordance with the American Society for Testing and Materials Standard Test Method for Flexibility of Running Shoes (ASTM F-911 - 85). The stamp was placed perpendicular to the longitudinal axis of the shoe. Each shoe was vertically displaced over 20 trials.

Output of the bending test (N and mm) are recalculated into bending moment (M) and bending angle (rad). Overall LBS was defined as the slope of the linear regression of the data points between 30 and 70% of the data of the moment-angle curve (Nm/rad; figure 3).


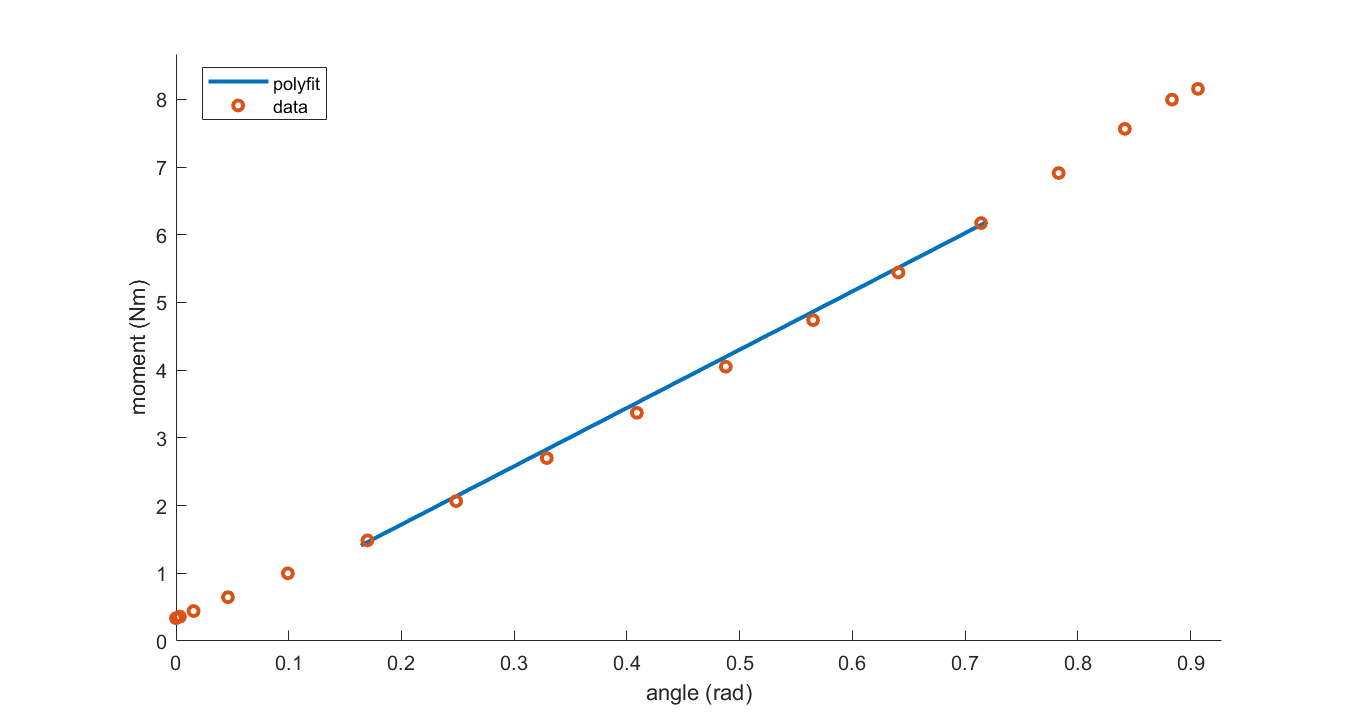


Figure 3. Moment-angle curve with linear regression between 30-70% of data

**Results**

The results of the paired samples t-tests showed that shoe stiffness increased significantly in the stiff sole condition for each of the three shoes (table 1).

| Table 1. Mean and std delta MTP angle, delta moment and shoe stiffness | | | | | | |
| --- | --- | --- | --- | --- | --- | --- |
|  |  | *Mean*  *Flexible sole* | *Mean*  *Stiff sole* | *t-value* | *df* | *p-value* |
| Shoe stiffness | Rocker size 39 | 26.8 | 47.0 | 17.45 | 4 | <0.01 |
| (Nm/rad) | No rocker size 40 | 18.2 | 102.2 | 25.44 | 4 | <0.01 |
|  | No rocker size 42 | 23.6 | 146.2 | 34.59 | 4 | <0.01 |
|  |  |  |  |  |  |  |

**References**

1. Oh K, Park S (2017) The bending stiffness of shoes is beneficial to running energetics if it does not disturb the natural MTP joint flexion. *J Biomech* **53**: 127–135.

2. Willwacher S, König M, Potthast W, Brüggemann G (2013) Does specific footwear facilitate energy storage and return at the metatarsophalangeal joint in running? *J Appl Biomech* **29**: 583–592.

3. Roy JPR, Stefanyshyn DJ (2006) Shoe midsole longitudinal bending stiffness and running economy, joint energy, and EMG. *Med Sci Sports Exerc* **38**: 562–569.
